# In situ structure and organization of the influenza C virus surface glycoprotein

**DOI:** 10.1038/s41467-021-21818-9

**Published:** 2021-03-16

**Authors:** Steinar Halldorsson, Kasim Sader, Jack Turner, Lesley J. Calder, Peter B. Rosenthal

**Affiliations:** 1 Structural Biology of Cells and Viruses Laboratory, The Francis Crick Institute, London, United Kingdom; 2 Present Address: Materials and Structural Analysis, Thermo Fisher Scientific, Achtseweg Noord 5, 5651 GG Eindhoven, The Netherlands

**Keywords:** Structural biology, Influenza virus, Cryoelectron tomography

## Abstract

The lipid-enveloped influenza C virus contains a single surface glycoprotein, the haemagglutinin-esterase-fusion (HEF) protein, that mediates receptor binding, receptor destruction, and membrane fusion at the low pH of the endosome. Here we apply electron cryotomography and subtomogram averaging to describe the structural basis for hexagonal lattice formation by HEF on the viral surface. The conformation of the glycoprotein in situ is distinct from the structure of the isolated trimeric ectodomain, showing that a splaying of the membrane distal domains is required to mediate contacts that form the lattice. The splaying of these domains is also coupled to changes in the structure of the stem region which is involved in membrane fusion, thereby linking HEF’s membrane fusion conformation with its assembly on the virus surface. The glycoprotein lattice can form independent of other virion components but we show a major role for the matrix layer in particle formation.

## Abstract (web summary)

Influenza C virus contains a single surface glycoprotein, the haemagglutinin-esterase-fusion (HEF) protein, that mediates receptor binding, receptor destruction, and membrane fusion activities. Here, the authors apply electron cryotomography of whole virus together with subtomogram averaging to determine the HEF structure and lattice organisation on the viral membrane and they discuss mechanistic implications for virus budding and membrane fusion.

## Introduction

Influenza C virus is a globally spread, intensely circulating human pathogen of the *Orthomyxoviridae* family. Infection typically causes a mild upper-respiratory tract illness that can progress to severe disease of the lower respiratory tract, particularly in young children^1–3^. The lipid-enveloped influenza C virus infects cells by binding receptors on the cell surface and enters the cell by receptor-mediated endocytosis. Following fusion of the viral membrane with the endosome membrane, the viral genome can enter the cytoplasm. During assembly, it acquires an envelope and surface proteins by budding through host cell membranes. Influenza C contains seven negative−sense genomic segments encoding seven structural proteins and two non-structural proteins. It encodes a single envelope glycoprotein, the hemagglutinin-esterase-fusion factor (HEF) which is a multifunctional protein that mediates receptor binding, receptor destruction, and membrane fusion^4–7^. In contrast, influenza A and B have eight genomic segments and encode two surface glycoproteins with receptor binding and membrane fusion mediated by the haemagglutinin (HA) and receptor destruction by the neuraminidase (NA). HEF is therefore unique amongst the ortho- and paramyxovirus glycoproteins in mediating all three functions. Another unique feature is that the single HEF protein forms a hexagonal lattice on the surface of the virion which may play a role in assembly^8,9^. Lattices persist as glycoprotein networks when proteolytically released from virus^8,10^ suggesting that they are mediated by glycoprotein interactions and glycoprotein contacts are observed in low-resolution negative stain reconstructions^9^. Lattices are observed on both spherical and filamentous particles, but the filamentous morphology is a property associated with the M1 protein which forms a layer beneath the membrane^11,12^.

Proteolytic cleavage of the HEF0 precursor, producing disulfide-linked subunits HEF1 and HEF2, is required for membrane fusion activity that is activated when the virus encounters low pH during host cell entry through the endosomal pathway^4^. HEF2 is anchored in the viral membrane by a C-terminal transmembrane region and contains an N-terminal hydrophobic fusion peptide. The X-ray crystal structure of the bromelain-released trimeric ectodomain of HEF showed that each HEF monomer consists of three functional domains^13^. HEF1 forms a receptor-binding domain (R) that recognizes receptors containing 9-O-acetyl sialic acid and an esterase domain (E) that functions as a receptor-destroying 9-O-acetylesterase. In addition, N-terminal and C-terminal segments of HEF1 combine with HEF2 to form the membrane fusion domain (F) (Supplementary Fig. 1).

Knowledge of the structural organization of the influenza C virus envelope is required to understand how HEF performs its multiple functions in the context of the virus and how HEF plays a role in virus self-assembly. Here we apply electron cryo-tomography to study the influenza C virus envelope and subtomogram averaging to the HEF glycoprotein, revealing the in situ conformation of the protein in the virus surface lattice. The conformation has implications for structural re-arrangements of the HEF protein that mediate membrane fusion. In addition, the matrix layer formed by the M1 protein and associated with the inside of the virus envelope plays a role in determining particle morphology and genome packaging.

## Results

### Electron cryotomography of influenza C virus

**Fig. 1:**
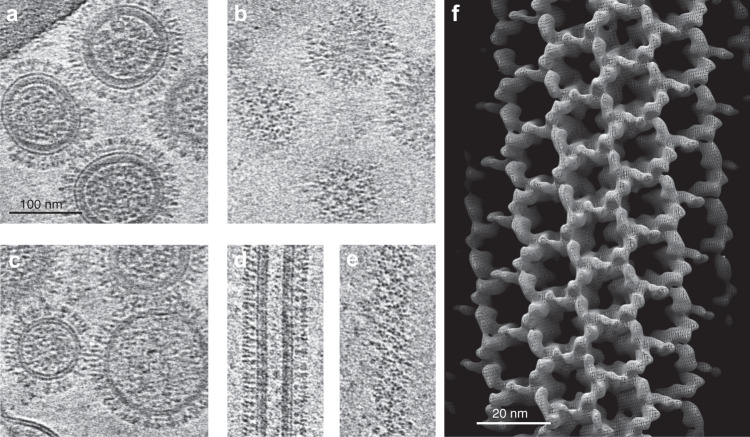
Electron cryotomography of influenza C virus. **a** A central section through spherical virions with a matrix layer. Visible are the outer glycoprotein layer on the virus surface, the electron-dense matrix layer that appears continuous with the inner membrane leaflet, and ribonucleoprotein particles (RNPs) in the interior. **b** The same virions as in panel **a** in a tomogram section showing the hexagonal organization of the glycoprotein layer on the virus surface. **c** A central section through particles lacking a matrix layer. The glycoprotein layer is visible but the matrix layer is absent beneath the bilayer and the interior is less electron-dense and lacks compact RNPs. These particles also exemplify the size variation in the particles without a matrix layer (See also Supplementary Fig. 2). **d**, **e** A filamentous particle is shown in a central section (**d**) or a section through the surface (**e**) showing the hexagonal organization of the glycoprotein layer. Each image in a-e shows a sum of 20 tomogram slices corresponding to a projected volume ~9 nm thick and are at the same scale. Tomograms were recorded (as described in Supplementary Table 1) from a single preparation of the virus. **f** Helical reconstruction from 2D images of a single filamentous virion at 32 Å resolution. The ~76 nm thick filament shows the hexagonal lattice covering the surface.

We purified viral particles and recorded cryo-EM images and electron cryo-tomograms of vitrified virions (Fig. 1a–e, Supplementary Fig. 2). The particles are pleomorphic and contain both spherical and filamentous particles of varying size covered by extended areas of the HEF glycoprotein organized in open hexagonal lattices, as previously described^9^. Analysis of tomograms in 3D reveals that spherical particles can be further classified by the presence (Fig. 1a, b) or absence (Fig. 1c) of a dense matrix layer in the virus interior adjacent to the inner membrane leaflet. Spherical particles with a matrix layer also contain a dense packing of ribonucleoprotein particles (RNPs) that package the genome in their interior. By contrast, spherical particles without a matrix layer have relatively empty interiors that lack RNPs but may contain small vesicles or cytoskeletal material. These empty particles suggest that the HEF glycoprotein layer on the membrane is sufficient for the budding of virus-like particles, in agreement with observations by electron microscopy of plastic sections of the plasma membrane of infected cells showing clustered surface projections and particles without RNP assemblies^10^.

Despite their similarity, we found a different size distribution for spherical particles with or without a matrix. We measured the maximum and minimum (in-plane) diameter of spherical-type viral particles from 31 tomograms (*n* = 220, Supplementary Fig. 3) and found particles with a matrix layer are more homogeneous, having a slightly smaller mean diameter and a much smaller variance (88.2 nm (STD 5.4) compared to 97.7 nm (STD 30.6)). The matrix layer is thus associated with and may impart a more defined size and curvature of the virus particles. Alternatively, because the matrix layer may be the point of attachment of the RNP assembly, the size of the packaged RNP assembly may also determine virion size and morphology.

Filamentous particles of varying diameter sometimes packaged RNPs at one end, but always contained a cylindrical matrix layer, usually surrounding a largely empty interior, suggesting a structural role for the matrix layer. The hexagonal glycoprotein lattice displayed in some cases a helical organization, but with varying symmetry between different particles. We obtained a low-resolution map (~32 Å) from several images of a single filament recorded at different defocus values showing the open hexagonal lattice (Fig. 1f) formed by HEF trimers.

### In situ structure of HEF by subtomogram averaging

**Fig. 2:**
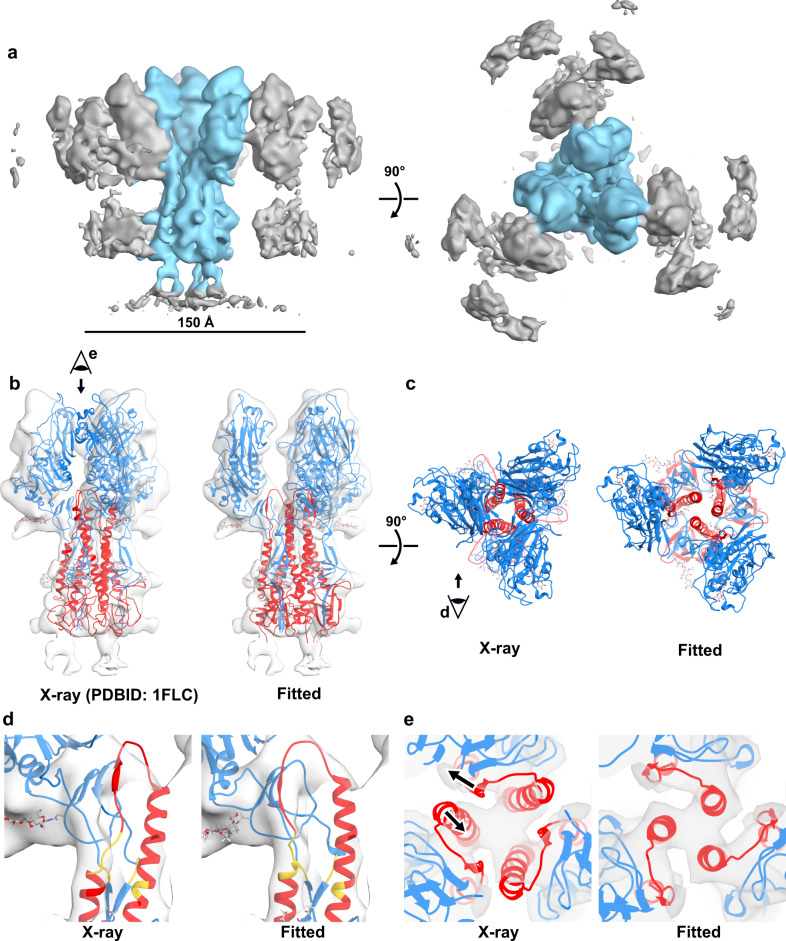
In situ structure of a HEF trimer based on subtomogram averaging. **a** Side (left) and a top view (right) of a map (9.2 Å resolution, Supplementary Fig. 4) obtained by averaging subvolumes containing HEF from the membrane of influenza C virions possessing a matrix layer. The central trimer is colored blue and the neighboring trimers and membrane are colored grey. **b** Atomic model fitting of the HEF trimer X-ray structure (left; pdbid:1flc) and the flexibly-fitted model into the HEF map (transparent surface) segmented to include only the central trimer (right). HEF1 is blue and HEF2 is red. **c** View down the trimer axis of the HEF X-ray structure (left; pdbid:1flc) and the flexibly fitted model (right) showing large displacements and 20-degree rotation of membrane distal HEF1 and association of the central helices at the top of HEF2. **d** A side view of a single monomer of HEF in the map as described for panel **b**. HEF1 residues 33–34 and 408–409 and HEF2 residues 65–69 and 99–100 are colored yellow where the hinge motion occurs. The interhelical loop between the HEF2 central helix and outer helices is shown, as well as the β-sheet it forms with HEF1. **e** A top view down the trimeric axis of the HEF map as described for panel **b**. The two arrows indicate the direction of displacement of the top of the central helix and the interhelical loop of HEF2 when comparing the crystal structure to the fitted model. See also Supplementary Movie 1.

To obtain more detailed structural information of the glycoproteins without dependence on long-range order in filaments, we applied subtomogram averaging to glycoproteins on spherical virus particles. We picked and averaged subtomograms of the envelope from spherical virus particles with a matrix, revealing the 150 Å long trimeric HEF (Fig. 2a) at 9.2 Å resolution (Supplementary Fig. 4, Supplementary Table 1). The map shows clear density for the central and outer alpha-helices in the membrane-proximal HEF2 stem region, but the membrane distal HEF1, which is mostly beta-sheet, has fewer resolved secondary structural features. Local resolution estimation indicates that the HEF2 stem region is generally better resolved, suggesting some flexibility in the HEF1 membrane distal region (Supplementary Fig. 5). Also visible in the map is the density for neighboring HEF’s at contact points on the HEF1 head region.

A comparison between the newly obtained map and the crystal structure of the trimeric ectodomain of HEF reveals a dramatic difference in the overall conformation of the molecule (Fig. 2b). The map and the X-ray model show good agreement in much of the stem region but the maps diverge in the head region where HEF1 domains are splayed out. We applied flexible fitting of the X-ray model to obtain a model for the in situ structure of HEF (Fig. 2b, c). Comparison^14^ of the in situ model with the X-ray model shows points of flexion in both HEF1 (residues 33–34 and 408–409) and HEF2 (residues 65–69 and 99–100). A model with a similar agreement to the map may be obtained by fitting each X-ray model monomer as 4 rigid bodies identified by the points of flexion. The resulting models have better agreement with the map as measured by cross-correlation and map-model Fourier Shell Correlation (cc score of 0.93, map-to-model FSC of 10.9 Å at 0.5, Supplementary Fig. 6). The HEF1 head region in isolation (residues 41 to 371 of HEF1) behaves as a rigid body and itself does not undergo any significant conformational changes in our fitted model compared to the X-ray structure (1.0 Å^2^ RMSD). The HEF1 head region rotates 20° clockwise (viewed toward the HEF2 stem) and its center of mass is displaced ~7 Å further away from the three-fold axis toward the peripheral density of the nearest neighboring trimer (Supplementary Video 1).

The large movement of the HEF1 head region is accompanied by changes in the organization of the long trimeric α-helices that form the central architecture of HEF2 (Fig. 2D, Supplementary Fig. 7). Similar to influenza A HA, HEF2 has long central alpha-helices along the three-fold axis and smaller N-terminal alpha helices connected by an interhelical loop and preceded by the N-terminal fusion peptide (Supplementary Fig. 1). However, the central helices of HEF2 in the X-ray structure interact closely in the middle but diverge from the trimer axis at the top (interhelix distance ~24 Å) where the interhelical loops interpose between them (Fig. 2c, e). Each interhelical loop of HEF2 also participates as an antiparallel strand in a four-stranded β-sheet that includes part of the esterase domain (HEF1 residues 378–398) (Fig. 2d, Supplementary Fig. 1). In the outward position of HEF1 observed in the in situ structure, the HEF2 interhelical loop remains associated with the HEF1 beta-sheet and no longer interposes between long helices (Fig. 2d, e) allowing the tops of the central helices to associate along the trimer axis (interhelix distance ~12 Å) (Fig. 2e and Supplementary Movie 1). The central helices in the in situ structure thus make interactions that are required to form an extended coiled-coil intermediate at low pH as observed for influenza A HA^15,16^.

In the in situ structure, we identify prominent elongated densities on the surface for carbohydrates at N-linked glycosylation sites N12 and N381 of HEF1 and N106 of HEF2 (Supplementary Fig. 8) that further validate the fitted model. In addition, we can tentatively assign density to N157 of HEF2. Approximately nineteen amino acids (residues 166–184) beyond the C-terminus of our fitted model make the connection to the transmembrane region. In this membrane-proximal region, we observe a bifurcation of the map density (Fig. 2a and Supplementary Fig. 8) and therefore require a higher resolution map in this region to interpret how the attachment to the membrane is made. Though the bottom part of HEF2 is similar in both the X-ray and in situ structures (Supplementary Fig. 7) and differences in the membrane distal parts reflect a clear hinge point 75 Å from the membrane, we cannot exclude that differences in the ectodomain structures result from the attachment of the transmembrane anchor in the viral envelope. For influenza A HA, the ectodomain is connected to the membrane by flexible linkers and the structure of the ectodomain is unchanged by the transmembrane anchor^17^.

### HEF forms a hexagonal lattice via HEF1–HEF1 contacts

**Fig. 3:**
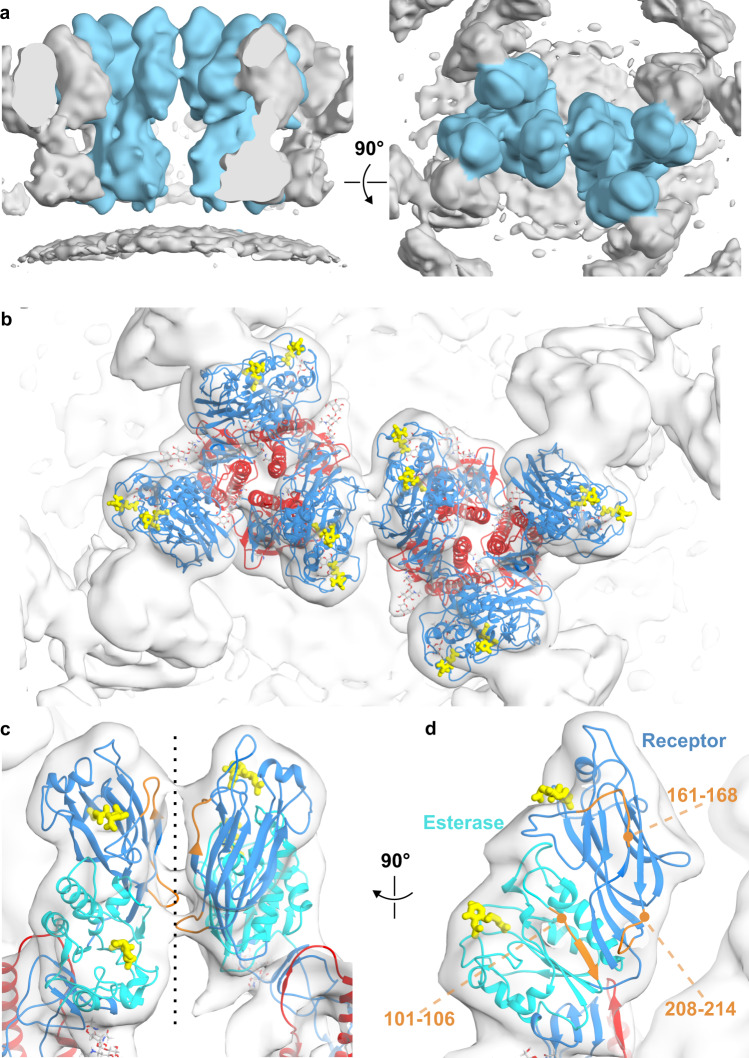
The HEF1-HEF1 dimer interface is the basis for lattice contacts between HEF trimers. **a** Side (left) and a top view (right) of a map (10.2 Å resolution, Supplementary Fig. 4) obtained by subtomogram averaging of two interfacing HEF trimers. The central dimer of trimers is colored blue and neighboring trimers and membrane are colored grey. **b** Two trimers of the fitted HEF model viewed down the two-fold symmetry axis. **c**, **d** The HEF1-HEF1 interface shown in more detail. HEF model shows receptor-binding domain (blue), esterase domain (teal), and HEF2 (red). Three polypeptide segments that are proximal in the dimer interface and may form homotypic contacts (HEF1 R domain loop 161–168) and heterotypic contacts (HEF1 R domain loop 208–214 and HEF1 E domain 101–106) are shown in gold. Potential homotypic contacts (containing atoms within 6 Å) between HEF1 R domain loops 161–168 include Leu164 (100% conserved) and Thr167 (99.7% conserved). Heterotypic contacts (containing atoms within 6 Å) occur between HEF1 R domain loop residues Gly211 and Thr212 (both 100% conserved) and HEF1 E residues Tyr101 (100%), Leu102 (100%), and Gln104 (100%), as well as Arg81 (99.4%), part of an additional highly conserved esterase segment. Conservation is based on NCBI non-redundant sequences for influenza C HEF. Panel **d** shows the view of the interface on one monomer after segmentation. Ligands (yellow) identify the receptor binding and enzyme active sites in **b**–**d**.

In the map of the HEF trimer in the hexagonal lattice, density for neighboring trimers was also evident. To further investigate the contacts involved in forming the hexagonal lattice, we applied subtomogram averaging on interfacing dimers of HEF trimers and obtained a 10.7 Å map (Fig. 3a, Supplementary Fig. 4) showing two well-resolved HEF trimers and contact points between HEF1 domains at their periphery. The in situ model obtained for the single HEF trimer fits well into the density of the two contacting HEF trimers when placed as rigid bodies (cc score of 0.92, map-to-model FSC of 13.6 Å at 0.5, Fig. 3b).

The in situ “open” conformation of HEF appears to be stabilized by HEF1-HEF1 interactions and the splaying of the membrane distal regions is required to mediate the contacts. We identify polypeptide segments in HEF that are proximal at the interface (Fig. 3c, d). While the esterase domain is at the most peripheral position in the X-ray ectodomain structure, the conformation observed here additionally brings the receptor-binding domains into proximity. The model for HEF interactions shows that the receptor binding sites and the esterase active sites are exposed within the lattice, with the receptor-binding site being closer to the apex. Antigenic epitopes identified by antibody escape mutants are predicted to be mostly exposed in the lattice, but antigenic sites A-3 (HEF1 residue 164) and A-4 (HEF1 residue 212) are located at interfaces between trimers in the lattice where binding may potentially disrupt lattice contacts^18,19^. The hemagglutinin-esterase (HE) occurs as a dimer on the surface of some beta coronaviruses and is structurally homologous to the R and E domains of HEF (25% sequence identity)^20^. We note that the HEF1-HEF1 interface between trimers in influenza C is different from the dimeric interface observed for the hemagglutinin-esterase (HE)^20^.

We also applied subtomogram averaging to spherical virions lacking a matrix layer and reconstructed maps of a single trimer and a dimer of trimers (Supplementary Fig. 9) to 10.0 Å and 11.3 Å resolution, respectively (Supplementary Fig. 4). The maps show that HEF adopts the same open conformation as we observed on spherical virions with a matrix layer and the in situ models of HEF fit well into the trimer map (CC score of 0.91, map-to-model FSC is 12.1 Å at 0.5). The model of a dimer of trimers also fits the map well (CC score of 0.95, map-to-model FSC is 12.9 Å at 0.5) with minor differences in density at the trimer interface. The HEF glycoproteins thus form the same lattice structure independent of the matrix layer and in absence of other virion components.

### Plasticity of the HEF lattice

**Fig. 4:**
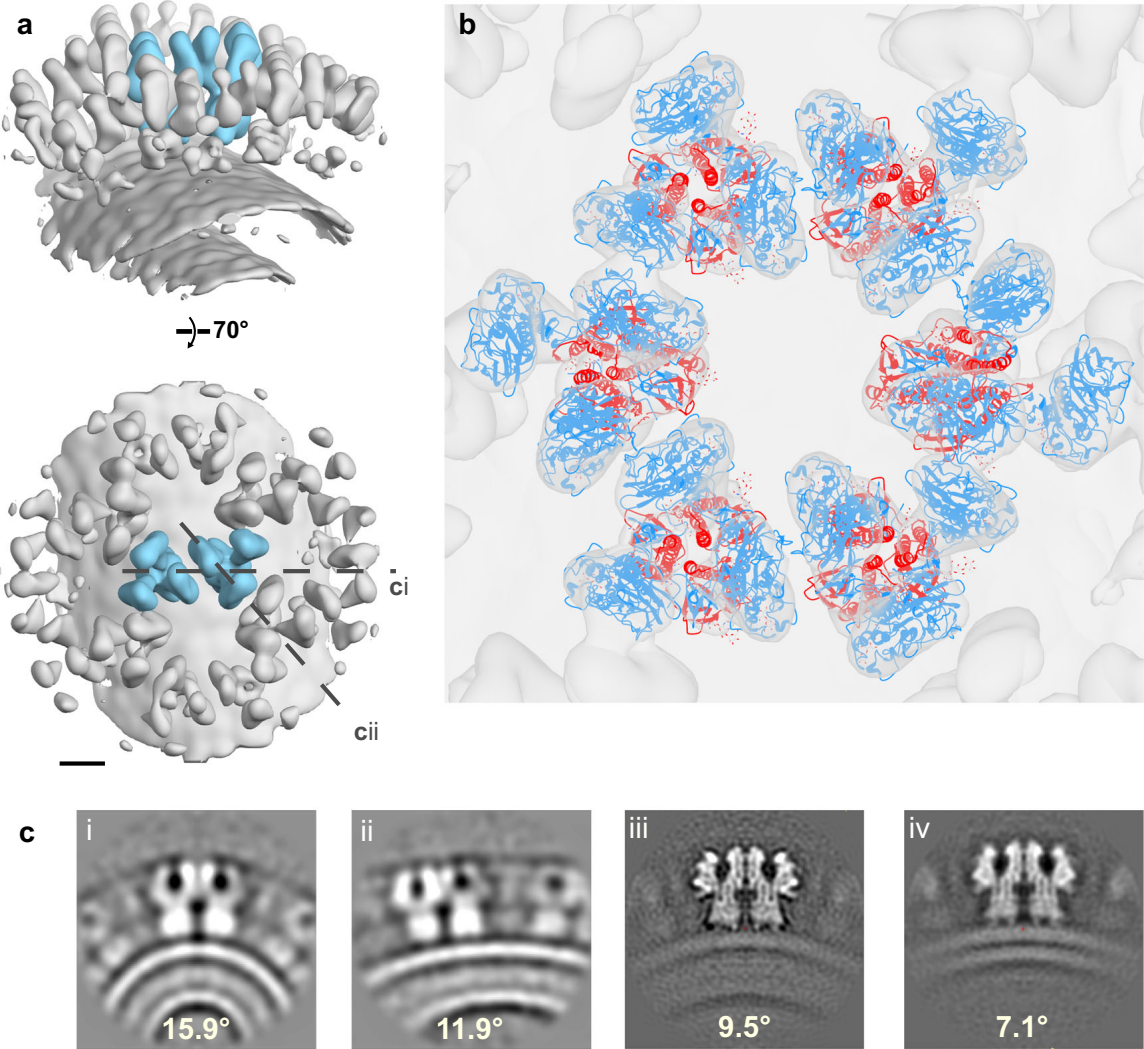
Plasticity of the HEF surface lattice. **a** Two views of a map obtained by subtomogram averaging of a region of a filamentous virion (26 Å resolution, Supplementary Fig. 4). Scalebar 50 Å. **b** Trimer models fit into the map in **a**. **c** Cross-sections through maps obtained by subtomogram averaging showing HEF interface on the membrane with different curvatures. Panels i and ii show sections through the filament map in panel **a** at lines **c**i and **c**ii, respectively. Panels iii and iv show dimers of trimers calculated for virions with (iii) and without (iv) a matrix layer visible beneath the bilayer. Angles between HEF 3-fold axes are indicated.

Subtomogram averaging of a filamentous particle was used to reconstruct a region of the HEF lattice (26 Å, Fig. 4a, Supplementary Fig. 4) showing that HEF adopts the same in situ conformation. We fit several HEF trimer models into the density on the cylindrical surface (Fig. 4b). As shown in sections through the map, the curvature on the membrane is greater on the filament than on the spherical particles and also depends on direction (Fig. 4c). The HEF trimers make dimer interactions with a relative angle between trimer axes of 11.9 and 15.9 degrees. While the cylindrical shape of the filament may largely be determined by the matrix layer, HEF can form lateral contacts at different membrane curvatures. In spherical particles with and without a matrix, the relative trimer angle between trimer axes is 9.5 and 7.1 degrees, respectively, the latter due to the slightly lower radius of curvature of the particles. The observed flexibility at the hinge regions of HEF1 may be a mechanism to adjust the dimeric contacts to different curvatures, though higher resolution analysis is required to understand the different contacts.

## Discussion

Our structural analysis of HEF by subtomogram averaging reveals the in situ conformation of the glycoprotein on the virus surface. The dramatic splaying of the membrane distal HEF1 creates HEF1-HEF1 dimeric contacts between trimers that are the structural basis for the hexagonal surface lattice. The same HEF conformation and lattice are observed on particles where HEF is present without the matrix layer and internal assembly of RNPs. We conclude that hexagonal lattice formation on the membrane is mediated by glycoprotein interactions and is not dependent on interactions between other virion components. The HEF lattice is plastic and accommodates a range of curvatures which may be important in driving assembly during the budding of the virus.

Secondly, though HEF lattices may form independently of the matrix layer, the matrix layer has an important role in virus particle assembly. The presence of the matrix layer influences the particle curvature and morphology as shown for the different particle types studied here. This is consistent with observations that single amino acid substitutions in M1 influence the formation of a filamentous particle type^21^ (“cords”) that typically lack RNP assemblies^12^. It is thus likely that interactions between the matrix and the envelope, as well as the attachment of the RNP assembly to the matrix layer, determine the uniformity of spherical particle sizes and shapes.

**Fig. 5:**
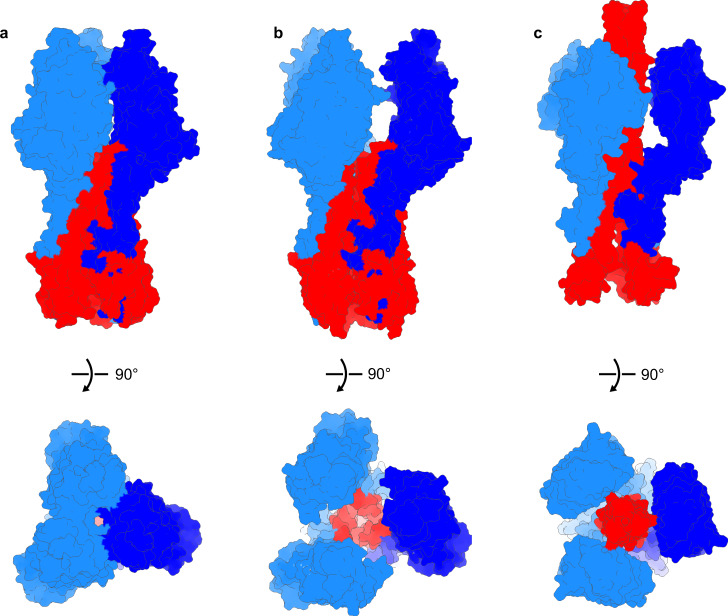
In situ structure of HEF has implications for structural rearrangements that mediate membrane fusion. **a** Closed conformation of ectodomain X-ray structure (pdb id: 1FLC). **b** In situ structure shows splaying of the membrane distal domains of HEF1 (blue/cyan) with an open cavity on the trimer axis revealing the HEF2 subunit (red). **c** Influenza A HA (pdbid: 6Y5K) extended intermediate shows that membrane distal HA1 subunits (blue/cyan) are displaced outward with the extended coiled-coil of the HA2 subunit (red) projecting between them. The top row shows a side view, the bottom row shows a view along the trimer axis looking toward the membrane.

The conformation of HEF on the virus surface has features important to the mechanism of membrane fusion. The re-positioning of the membrane distal domains of HEF1, which is the basis of the lattice contacts, is coupled to a structural change in HEF2 that brings the top of the long, central helices closer along the trimer axis. The in situ arrangement may facilitate the N-terminal extension of a HEF2 coiled-coil at the pH of membrane fusion. In addition, the splaying of the head domains creates an open cavity along the trimer axis that exposes HEF2 (Fig. 5) and is sufficiently wide to accommodate a HEF2 extended intermediate similar to that described for influenza A HA that moves the fusion peptide toward the target membrane^16,22^. Indeed, a separation of the membrane distal domains has been noted as a requirement for membrane fusion^23^ in influenza A HA and is observed by cryo-EM in structural transitions in HA at low pH before the formation of an extended intermediate^16^. Thus, the open HEF conformation in the surface lattice resembles an early dilated HA fusion intermediate and may similarly facilitate the low-pH transition and interaction with the target membrane. Lattice disassembly, observed following incubation of influenza C virus at low pH^5^, is likely to be a requirement for subsequent steps in membrane fusion where several HEF molecules bring the membranes together.

HEF is also present as the single surface glycoprotein on the orthomyxovirus influenza D (55% sequence identity)^24^ and is reported to form a hexagonal lattice on the virus surface^25^. The crystal structure of the HEF ectodomain from influenza D shows the same closed HEF conformation observed for influenza C. The closed conformation of HEF may be functionally important and may stabilize the pre-fusion state before HEF is integrated into the lattice. A function of HEF that may be essential before virus assembly is complete is the receptor-destroying esterase activity. The esterase activity is present on the assembled virus^7^ and may play a role in virus entry but is essential for hydrolyzing receptors on the cell surface and on virus glycoproteins to facilitate virus release from the cell surface during budding^26^. Mobility of HEF may be important for the esterase active site to reach and hydrolyze receptor molecules before HEF becomes constrained in the surface lattice and primed for the structural re-arrangements associated with membrane fusion. Our structural analysis of HEF on the virions shows the structural basis for lattice formation by HEF and that HEF functions involved in virus entry may be linked to HEF’s conformation in the lattice.

## Methods

### Virus growth, purification, and vitrification

Influenza C/Johannesburg/1/1966 strain was cultured in MDCK cells. Cells were maintained at 37 °C with 5% CO_2_ in DMED media supplemented with 5% fetal calf serum and penicillin/streptomycin in T175 triple-stacked flasks. Cells were washed in PBS and then infected in serum-free DMEM media supplemented with 0.02 M HEPES (pH 7.5), 2.5 μg/ml trypsin, and virus seed. The infection took place at 33 °C for 48 h. The cell supernatant was initially clarified by centrifugation at 650 × *g* in a benchtop centrifuge followed by ultracentrifugation at ~100,000 × *g* at 4 °C to pellet the virus. The virus was resuspended in Tris-buffered saline supplemented with 10 mM CaCl_2_ (TBS-Ca). The resuspended virus was either prepared for microscopy by plunge freezing (for 2D imaging of filamentous particles) or further purified by ultracentrifugation through a discontinuous 30–60% sucrose gradient at ~100,000 × *g* at 4 °C. The 30–60% interface was collected and diluted in TBS-Ca and finally pelleted again by ultracentrifugation at ~100,000 × *g* at 4 °C and resuspended in TBS-Ca. The virus was prepared for cryo-EM by vitrification using the Vitrobot™ (Mark III) on Quantifoil R 2/4 holey carbon grids with a 100 × 400 mesh that were glow-discharged in the presence of amylamine. The sample was mixed with 10 nm colloidal gold particles as fiducial markers prior to vitrification.

### Electron microscopy, image processing, and tomogram reconstruction

Data collection parameters and refinement statistics can be found in Supplementary Table 1. Cryomicrographs for helical reconstruction were collected on a Tecnai Spirit transmission electron microscope (TEM, FEI) operated at 120 keV and equipped with an Eagle 4k detector (FEI) at a pixel size of 3.6 Å/pixel. Tilt series for tomographic reconstruction and subtomogram averaging were collected on a Titan Krios TEM (Thermo Fisher) using the Tomography software (Thermo Fisher) for automation. The microscope was fitted with a Gatan GIF Quantum energy filter operating in zero-loss mode with a slit width of 20 eV and a K2 Summit direct electron detector (Gatan) operated in counting mode. Bi-directional tomographic tilt series were collected from 0° to +54° and −3° to −54° (or −42°) at a 3° interval in movie mode at a pixel size of 2.2 Å/pixel. Four frames were collected per tilt with a dose of 1.57 e^−^/Å^2^ per tilt, giving a total accumulated dose of 58.1 e^−^/Å^2^ (or 51.8 e^−^/Å^2^) for each tilt series. Movie frames were motion-corrected and dose weighted using alignframes from the IMOD package^27,28^. Tilt series were aligned using Etomo from the IMOD package^27,28^, the contrast transfer function (CTF) was estimated using CTFFIND4^29^ and tomograms were CTF corrected and reconstructed using novaCTF^30^.

### Helical reconstruction

A single filamentous virion was imaged at three different defoci on a Technai Spirit TEM (see above). Iterative helical reconstruction^31^ was performed using SPIDER^32^ where parameters for the helical rise (42.12 Å), turn (−18.53°), and symmetry (C7) were obtained. Further helical processing^33^ was performed in RELION 3.0^34^ where the filament was boxed, extracted and initial 2D averages were obtained. Further 3D real-space helical classification and refinement finally produced a structure of the hexagonal HEF lattice at ~32 Å resolution. The images were CTF corrected using CTFFIND4^29^.

### Subtomogram averaging (STA)

Tomograms containing viral particles decorated with glycoproteins were selected for STA. Spherical particles were grouped and processed separately based on the presence or absence of a matrix layer. Dynamo^35^ was used to pick coordinates of HEF on each viral particle surface by generating random seeds at either a 30 or 40 Å interval. 3D particles were extracted with trimvol from the IMOD package^27,28^. All subsequent steps were performed using RELION 3.0^34^. Initial processing was performed on tomograms that were binned by a factor of 2. Extracted particles were normalized before an initial round of 3D refinement with a cylindrical mask. Here a starting model was used that was generated from the crystal structure of the trimer (PDBID: 1FLC) using pdb2mrc from EMAN2^36^ and low pass filtered to 60 Å. Particles were then subject to a 3D classification using initially C1 or C3 symmetry and subsequently C3 symmetry. The resulting classes were only selected for further refinement when a clear elongated structure was observed, which was in all cases either one or two classes. Overlapping particles were removed and remaining particles were split into two different subsets where subtomograms from the same tomograms were grouped together to avoid aligning systematic noise. These two subsets were then used for a gold-standard 3D refinement and post-processing using a mask generated from a low pass filtered and segmented structure of the HEF from the previous 3D classification using Chimera^37^. Refined particles were then re-extracted from un-binned tomograms and subject to 3D classification, overlapping particle removal, regrouping, a gold standard refinement, and post-processing using C3 symmetry. The masks that were used for 3D classification, refinement, and post-processing of the un-binned maps were generated from a segmented structure (program Chimera^37^) of the HEF from a 3D classification that was low pass filtered and extended (at least eight pixels) and further given a soft edge (eight pixels) using the program relion_mask_create. In the cases of dimers of HEF trimers, a starting model was generated by using the calculated map of the single trimer to orient two crystal structure models of the HEF relative to each other by using the location of neighboring trimers which are visible in the single trimer map. These two models were then converted to a density map and low pass filtered to 60 Å. 3D classification, overlapping particle removal, regrouping, a gold standard refinement, and post-processing were performed using C2 symmetry with masks created as described above.

An initial 3D map generated de novo in Relion using stochastic gradient decent and subsequently refined showed the same features as refinements starting from the low-pass filtered X-ray model. In addition, a single filamentous virion was processed independently for the structure presented in Fig. 4a using a starting model of four trimers and later a mask containing 6 trimers.

### Viral particle measurement

The diameter of 220 viral particles from 31 tomograms was measured in IMOD^28^. Two measurements were taken from each particle, the maximum and minimum diameter in a horizontal plane. 136 particles with a matrix layer and 84 particles without a matrix layer were measured and the data were analyzed and plotted with the Seaborn package in Python. A Welch T-test and Levene’s test were performed with the SciPy package in Python and used to compare the mean and the distribution, respectively, of the particle populations.

### Flexible fitting of HEF crystal structure into EM density

The Molecular Dynamics Flexible Fitting (MDFF) tool^38^, a part of the VMD 1.9.3 package^39^, was used to generate a fitted model from the HEF crystal structure^13^ using the STA map of HEF from virions containing a matrix layer. All calculations were performed with NAMD 2.13^40^ using the CHARMM36 force field^41^. Simulations were performed in an explicit solvent with 0.1 mM NaCl, a Langevin piston algorithm was used to maintain constant pressure at 1.01325 bar and at a temperature of 293 K. Periodic boundary conditions and grid spacing of 3 Å were used. Non-bonded interactions were calculated every 2 fs and full electrostatics every 4 fs. Symmetry restraints were applied to the trimeric structure, glycans were restrained as rigid bodies and additionally, secondary structure, hydrogen bonds, cis-peptides, and chiral centers were restrained. A scaling force of 0.3 kcal/mol was applied to the density map. The simulations were run for 5 ns and cross-correlation scores were used to assess the fitting. Glycans were rebuilt post flexible fitting using Coot^42^ and the final structure was subject to one round of energy minimization with MDFF. DynDom was used to analyze and identify the hinge region in the fitted model. Results similar to the MDFF model were obtained using a rigid body fitted model consisting of two rigid bodies: (1) HEF1 residues 35–407 and HEF2 residues 70–98 (2) HEF1 residues 1–32 and 410–427 and HEF2 residues 6–64 and 101–160. Map-to-model FSC curves were computed using the *Phenix* package^43^. The local resolution of the map of the HEF trimer from spherical particles with a matrix layer was calculated using cryoSPARC^44^.

### Reporting summary

Further information on research design is available in the Nature Research Reporting Summary linked to this article.

## Supplementary information

Supplementary Information

Description of Additional Supplementary Files

Supplementary Movie 1

Reporting Summary

## Data Availability

Maps and models have been deposited in the Electron Microscopy Data Bank (http://www.ebi.ac.uk/pdbe/emdb/) under accession numbers “EMD-10810”, “EMD-10811”, “EMD-10812”, “EMD-10813”, and “EMD-10814”. A model has been deposited in the Protein Data Bank (https://www.ebi.ac.uk/pdbe/), under accession number 6YI5 [10.2210/pdb6YI5/pdb].
