# Supplementary material for: In situ structure and organization of the influenza C virus surface glycoprotein
Source: Nat Commun. 2021 Mar 16;12:1694. doi: 10.1038/s41467-021-21818-9 (PMC7966785; doi:10.1038/s41467-021-21818-9)
Supplement: Supplementary file 1 — Supplementary Information [file 41467_2021_21818_MOESM1_ESM.pdf]

Supplementary Information

In situ structure and organization of the influenza C virus surface glycoprotein

Steinar Halldorsson *et al*,

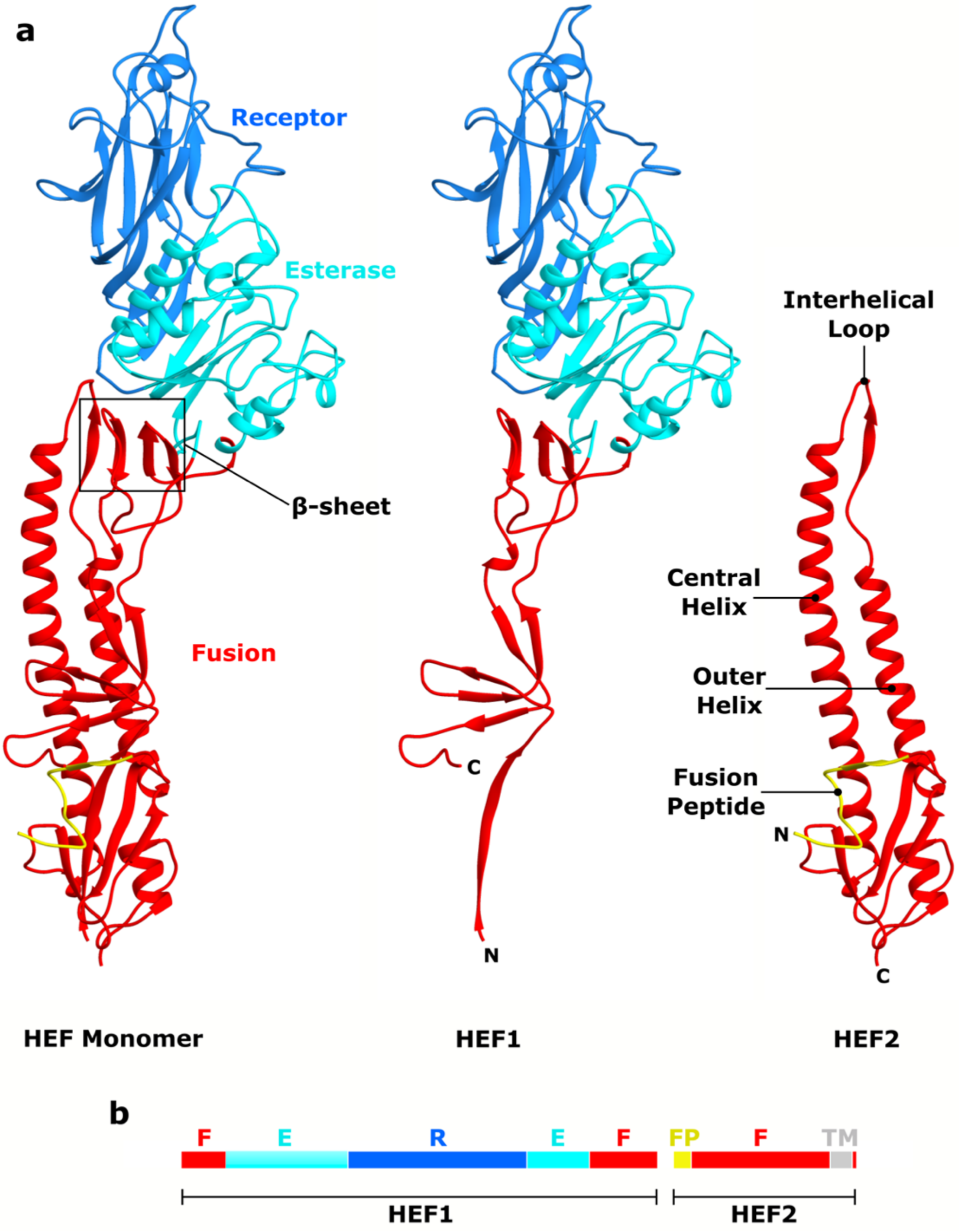

Supplementary Figure 1. Structure of the influenza C virus HEF ectodomain

a) Left: monomer model from the trimeric ectodomain structure determined by X-ray crystallography (pdbid: 1flc) coloured by domain: receptor binding domain (blue), esterase domain (teal), and membrane fusion domain (red). Middle: model of HEF1 subunit only. Right: model of HEF2 subunit only. Indicated are the long central and small outer helices of HEF2 and the β-sheet including 3 strands of HEF1 and a strand from the interhelical loop of HEF2. The N-terminus of HEF2 containing the fusion peptide (FP) is coloured yellow. **b)** Linear sequence segments of HEF coloured by domain as in panel a for R (receptor binding), E (esterase domain) and F (membrane fusion). HEF1 and HEF2 subunits are indicated. HEF2 is anchored by the transmembrane region (TM) at its C-terminus and has an N-terminal hydrophobic fusion peptide (FP).

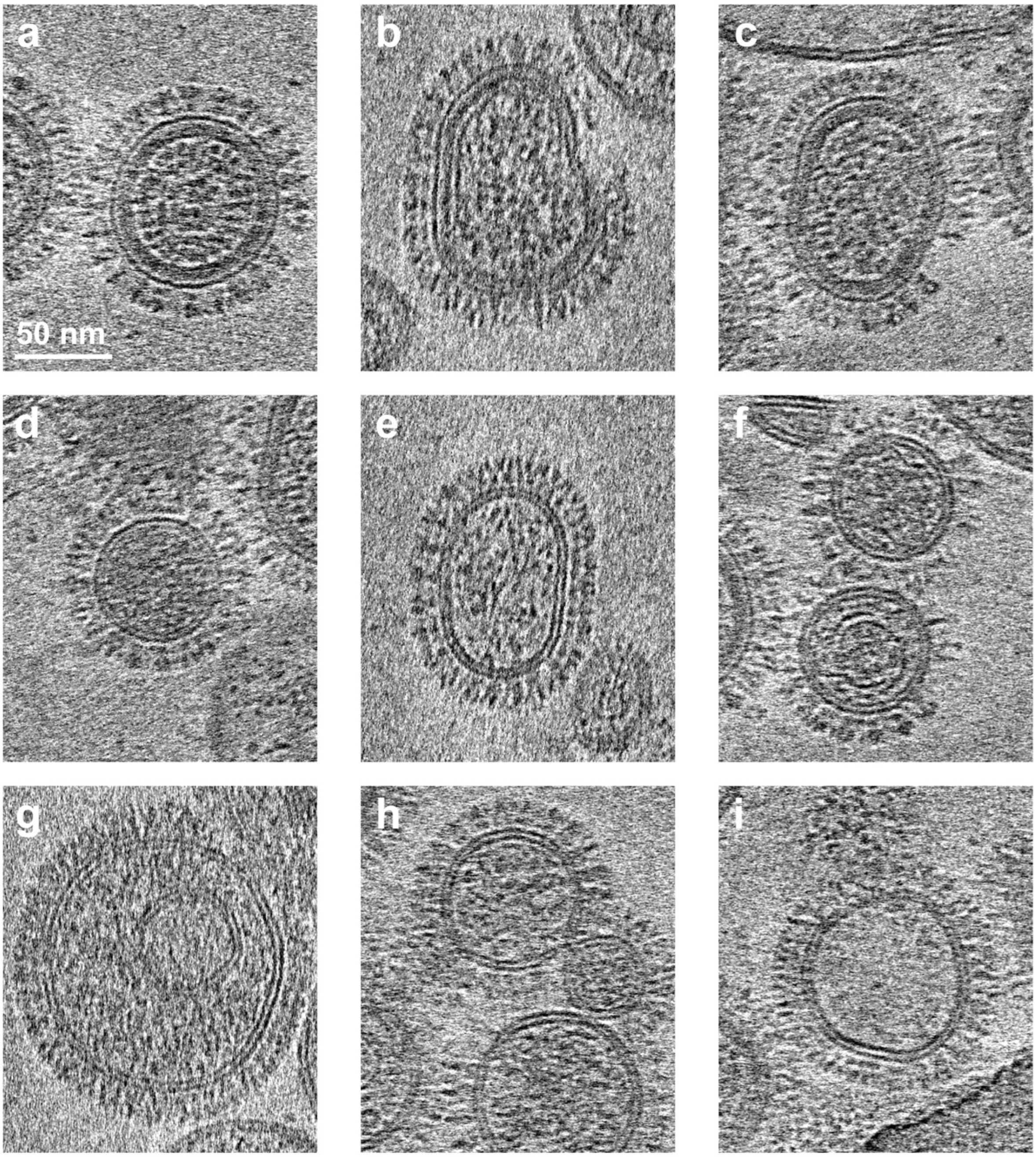

Supplementary Figure 2. Further examples of influenza C morphology
a-c) Representatives of particles with a matrix layer. Rounded particles with organised RNPs as in panel a are most commonly observed, but occasionally elongated particles are observed as in panel b and c**. d-i)** Representatives of particles without a matrix layer. A broad range of sizes and shapes is observed. The internal content also varies from crowded as in panel d to empty as in panel i, some even contain small membrane bound vesicles as in panel g. All examples are from 220 particles identified in tomograms recorded of a single virus preparation. Images are at the same scale and are sums of 20 tomogram slices corresponding to projected volume ~9 nm thick.

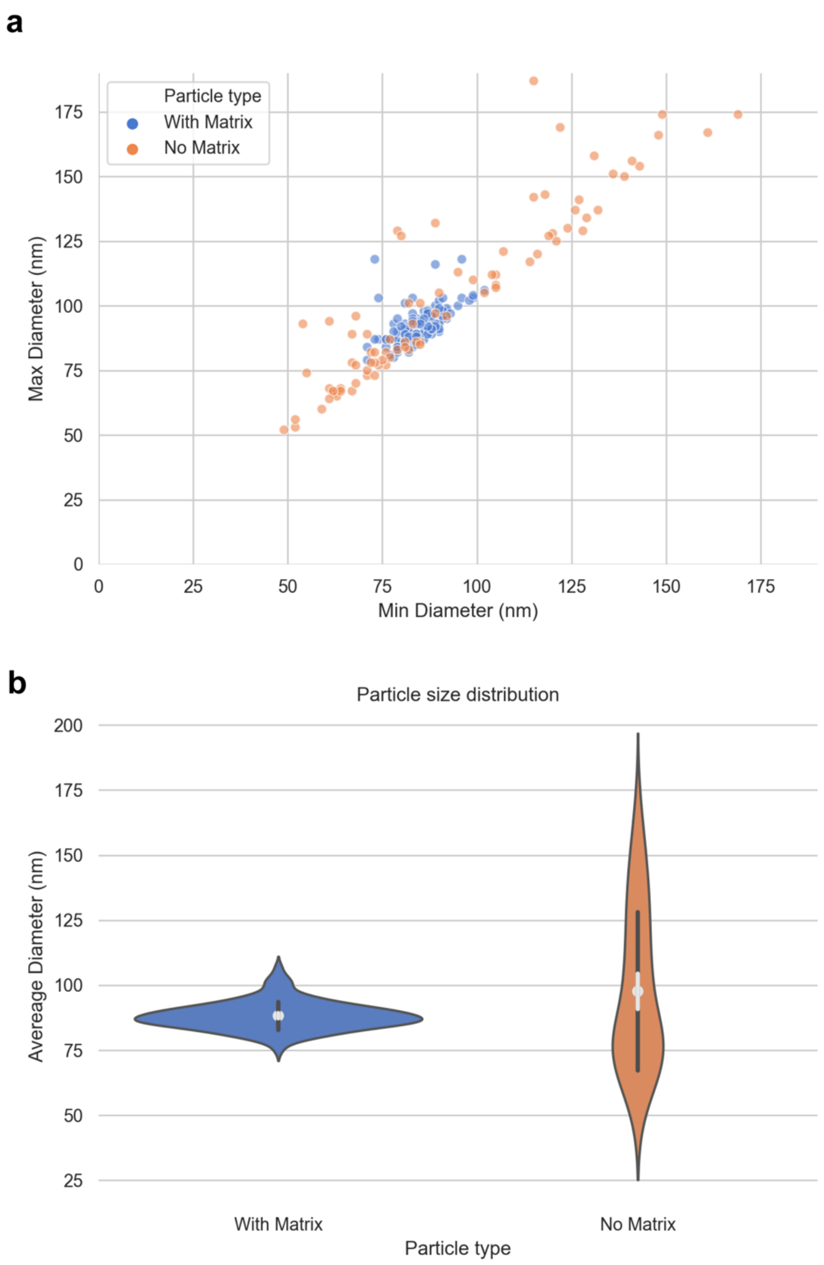

Supplementary Figure 3. Influenza C particle size distribution.

a) Scatter plot of minimum and maximum diameter of spherical particles in 31 tomograms recorded of a single virus preparation. Particles were scored for the presence (blue coloured points, n=136) or absence (terracotta coloured points, n=84) of a matrix layer. **b)** Violin plots for the average particle diameter (average of minimum and maximum diameters measured in tomogram central section) for particles in panel a indicating the population mean (grey dot), the 99% confidence interval (CI) of the mean (grey bar), and standard deviation (STD, black bar). For particles with a matrix layer (blue): Mean 88.2 nm, STD 5.4 nm and CI 87.2-89.2 nm. For particles without a matrix (terracotta): Mean 97.7 nm, STD 30.6 nm and CI 91.0-104.3 nm. The mean diameter of the two populations is significantly different (two-sided Welch's T-test: p=0.0068) and the distribution of the two populations is significantly different (two-sided Levene's test, $p=4.23 \times 10^{-24}$).

a

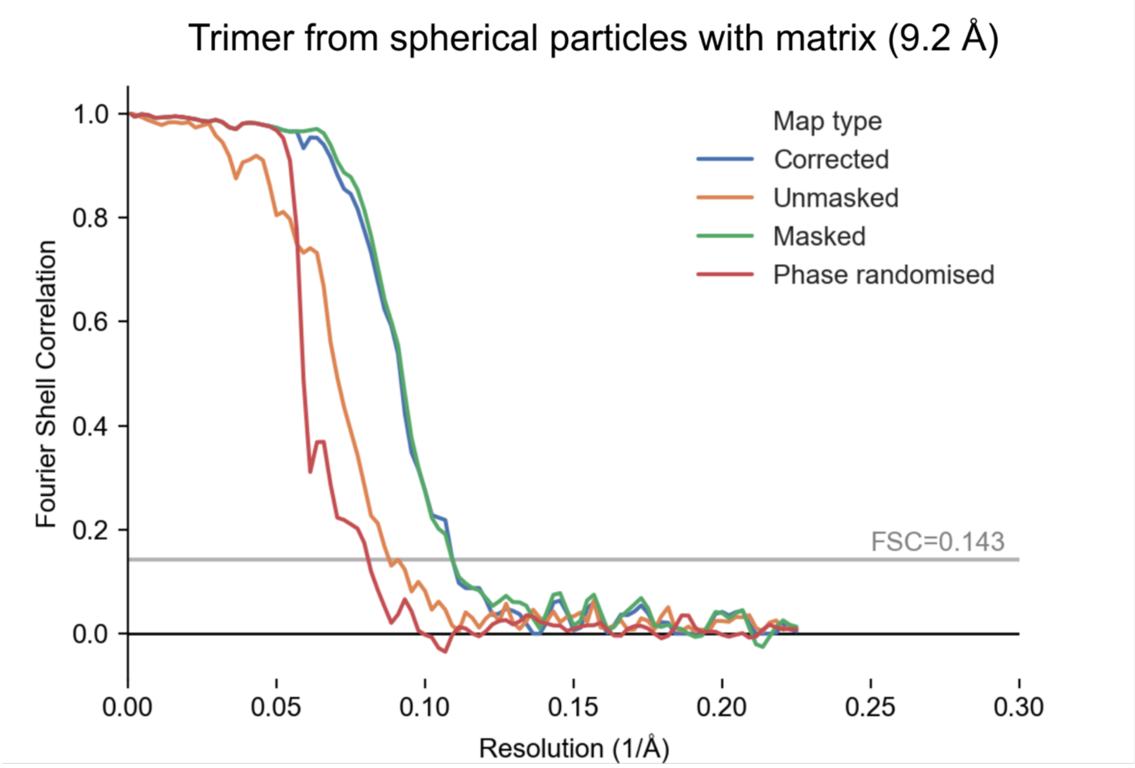
Trimer from spherical particles with matrix (9.2 Å)
Map type
Corrected
Unmasked
Masked
Phase randomised
FSC=0.143
Fourier Shell Correlation
Resolution (1/Å)
1.0
0.8
0.6
0.4
0.2
0.0
0.00
0.05
0.10
0.15
0.20
0.25
0.30

b

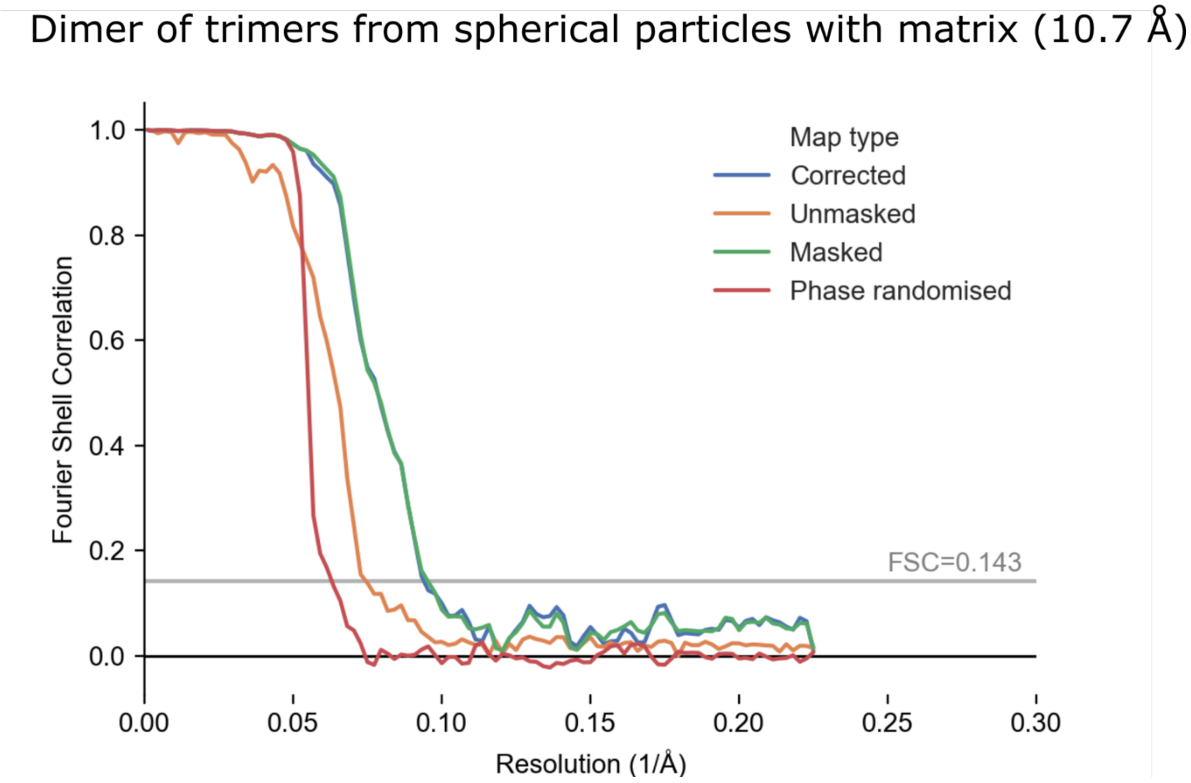
Dimer of trimers from spherical particles with matrix (10.7 Å)
Map type
Corrected
Unmasked
Masked
Phase randomised
FSC=0.143
Fourier Shell Correlation
Resolution (1/Å)
1.0
0.8
0.6
0.4
0.2
0.0
0.00
0.05
0.10
0.15
0.20
0.25
0.30

c

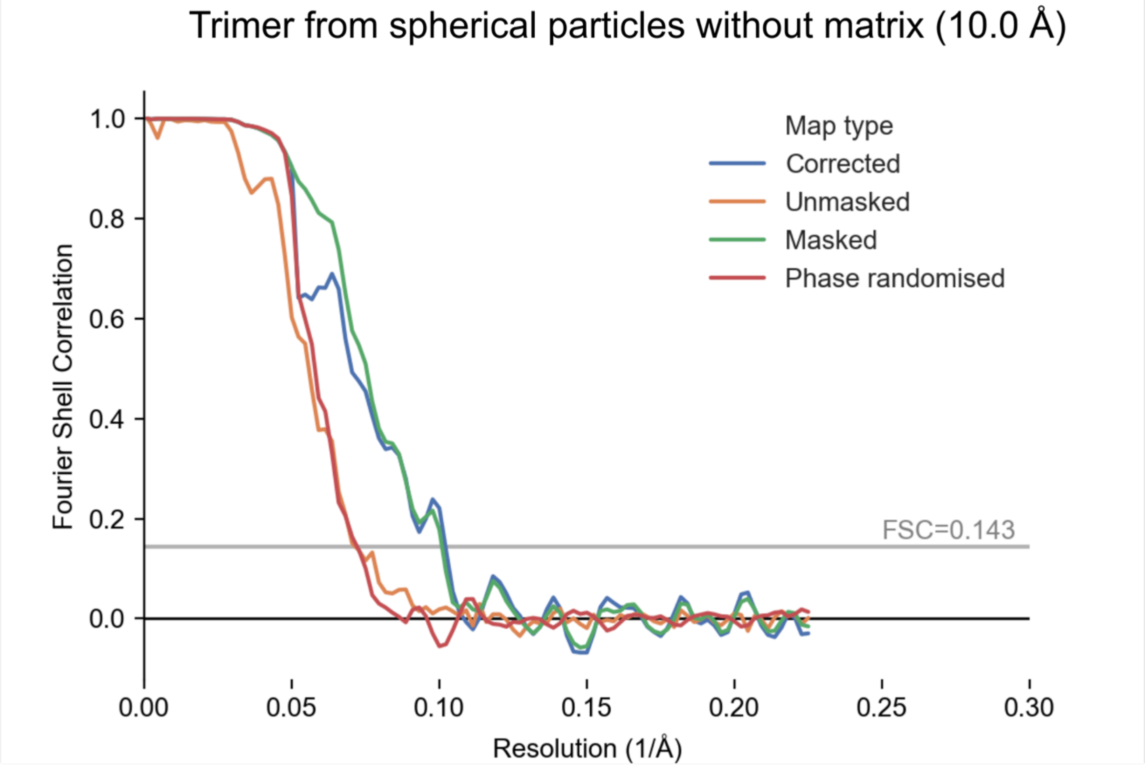

d

Dimer of trimers from spherical particles without matrix (11.3 Å)

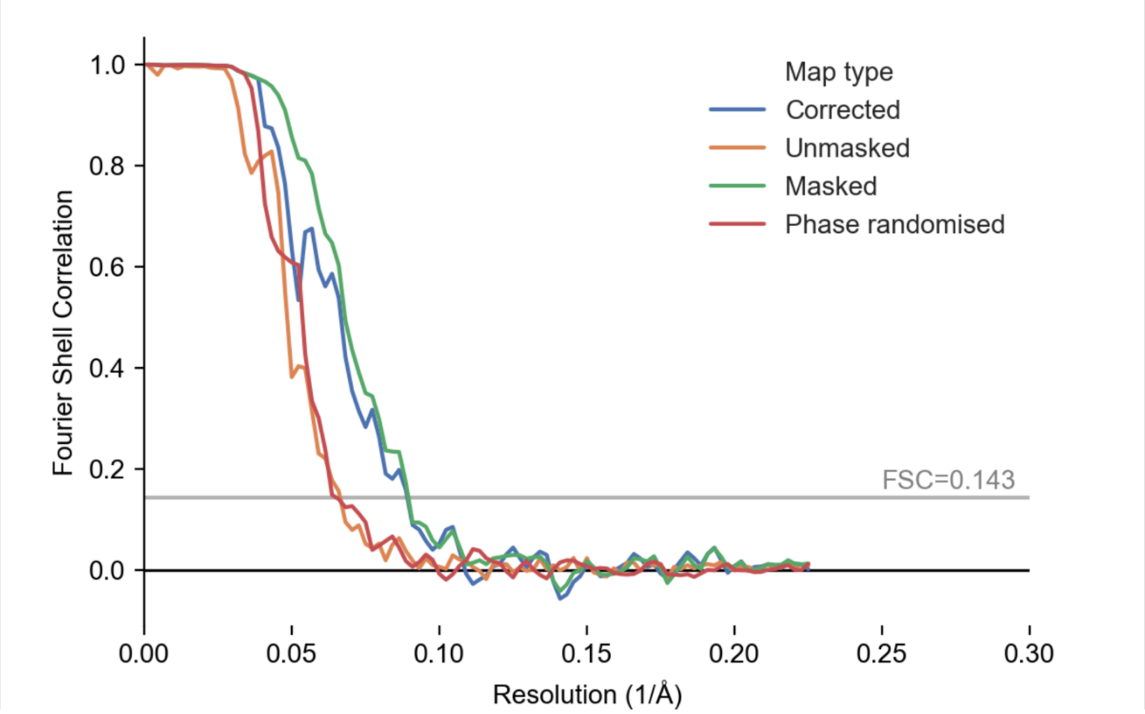

e

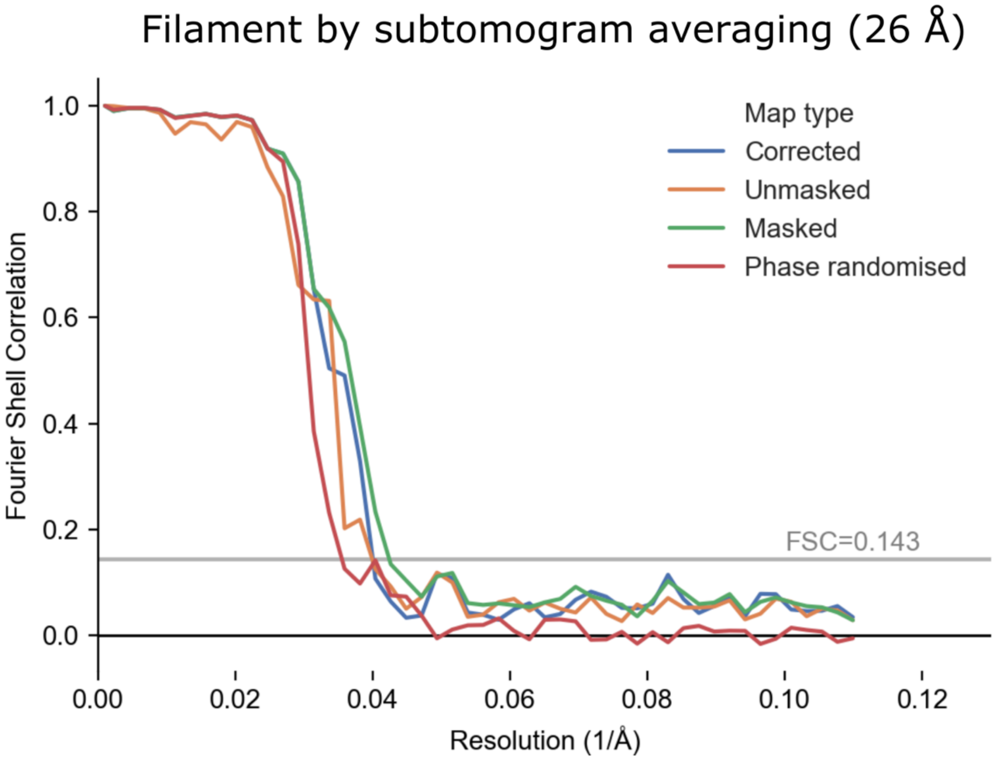

Supplementary Figure 4. Fourier Shell Correlation plot for all structures.
Curves are given for the unmasked, masked, phase randomised, and corrected calculations performed in Relion 3. Resolution is assigned at the FSC=0.143 threshold for the corrected curve. Curves in a, b, and e have small nonzero FSC values beyond the nominal resolution assignment.

a) Trimer from spherical particles with matrix layer indicating resolution of 9.2 Å.
b) Dimer of trimers from spherical particles with matrix indicating resolution of 10.7 Å.
c) Trimer from spherical particles without matrix layer indicating resolution of 10.0 Å.
d) Dimer of trimers from spherical particle without matrix layer indicating resolution of 11.3 Å.
e) Four tetramers (see Methods) from a filamentous particle indicating a resolution of 26 Å.

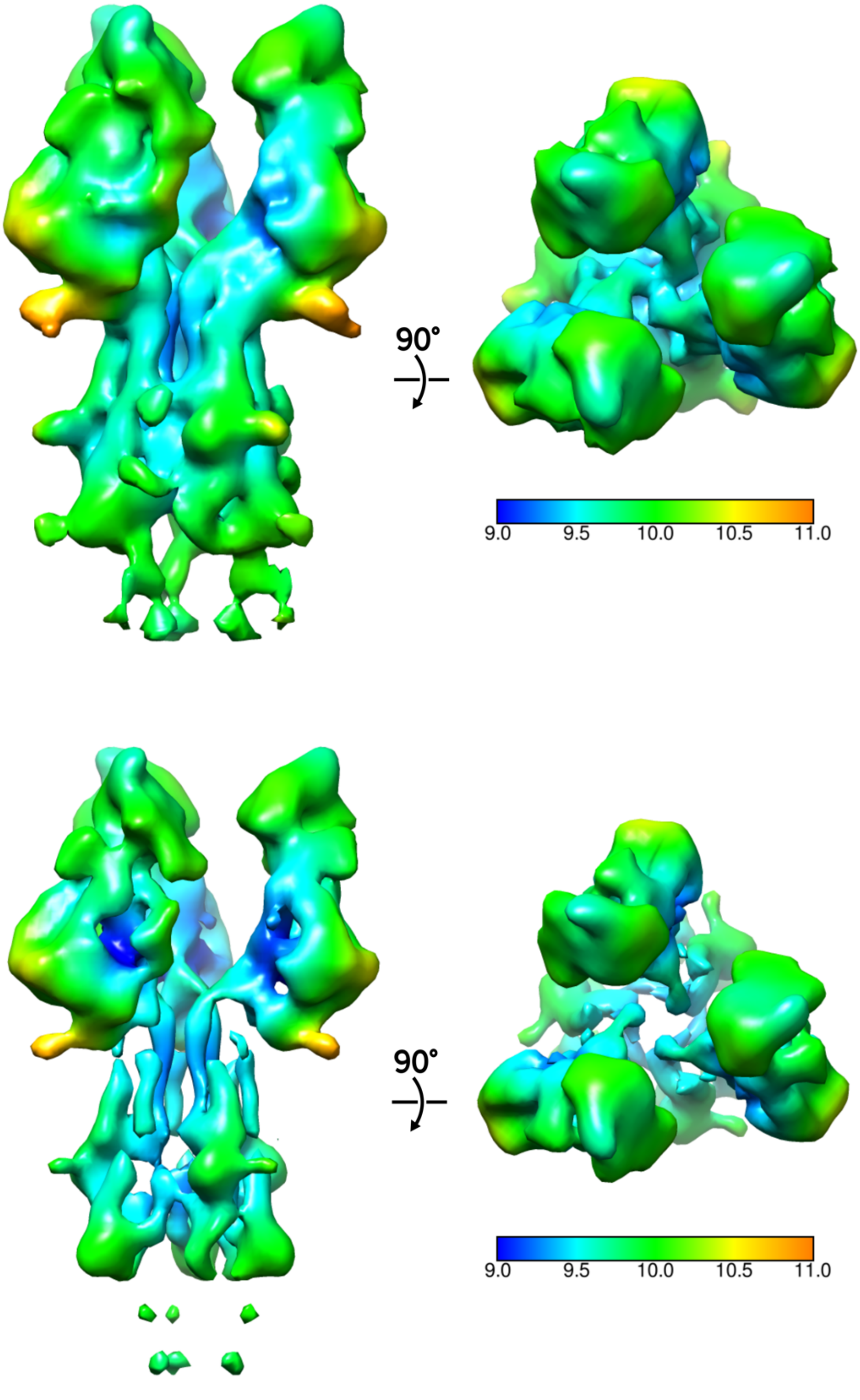

Supplementary Figure 5. Local resolution map of the HEF trimer.
The local resolution of the map of the HEF trimer from spherical particles with a matrix layer was calculated using cryoSPARC. A segmented density of the individual trimer is shown in side and top views at two different map contour levels. The map is coloured according to resolution (Å) as indicated by the colour key. The upper panel shows a medium contour level and the lower panel shows a higher contour level.

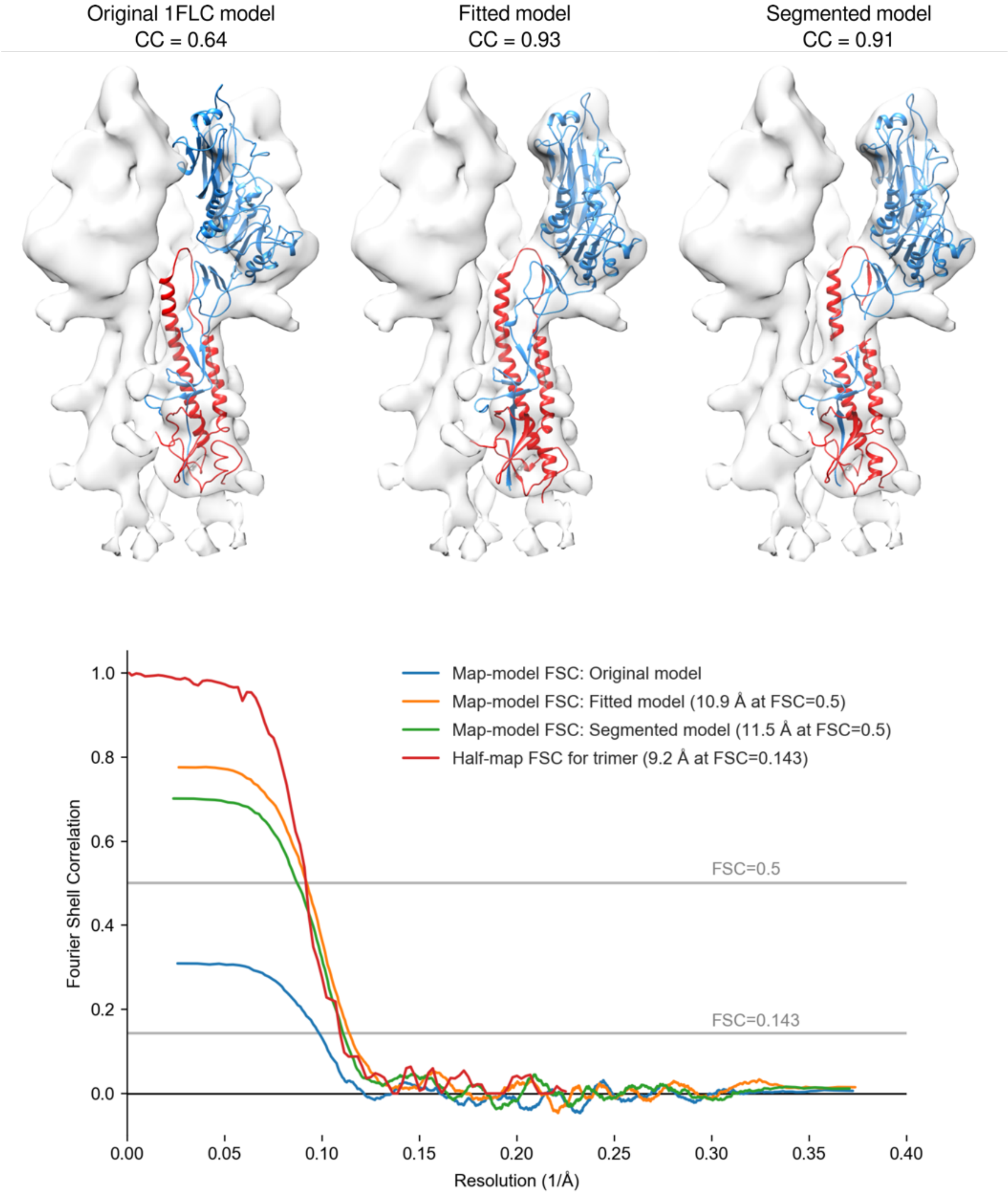

Supplementary Figure 6. Model fitting quality estimation.
The quality of structural models of HEF were estimated by map-model cross-correlation (CC) scores and Fourier shell correlation (FSC). The top panels show single HEF monomers from each of the models inside the map density and the associated CC scores. The graph plots the FSC curve between half maps and the map-model FSC curves (*Phenix* package) for the X-ray structure of HEF (pdbid: 1FLC) and models obtained by flexible fitting or as rigid bodies (see **Methods**).

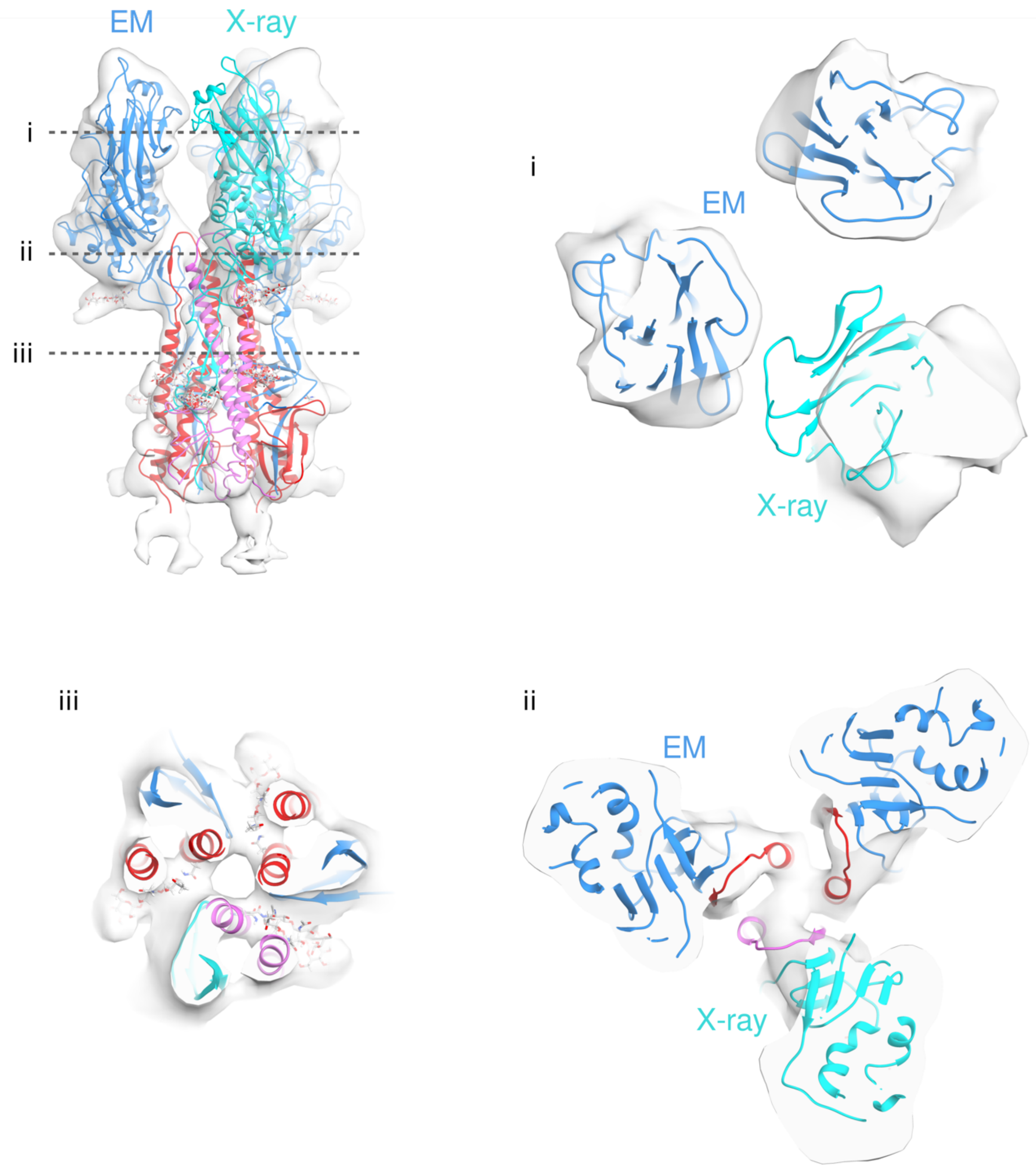

Supplementary Figure 7. Density features and model fitting.
Model fit to trimer map from particles with matrix. Two EM fitted HEF monomers are shown in blue (HEF1) and red (HEF2) and a single pre-fitted HEF monomer (X-ray structure, pdbid: 1FLC) is shown in cyan (HEF1) and pink (HEF2). Sections through the map are shown at (i) HEF1 head domains (ii) top of HEF2 central helices and interhelical loop and (iii) central helices and small outer helices. Locations for slices are show in panel at top left.

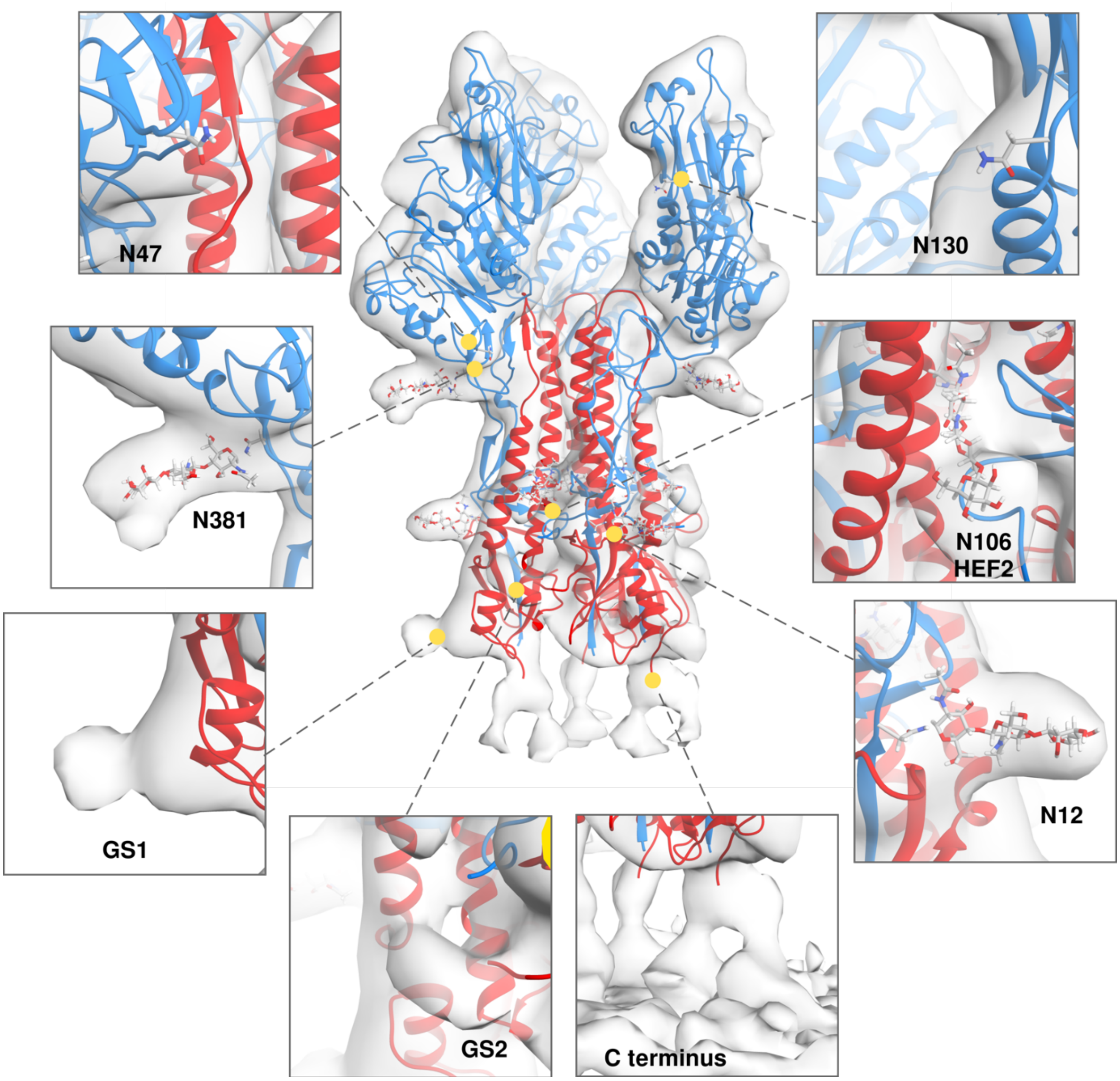

Supplementary Figure 8. Glycosylation sites and additional features in HEF map.

The trimeric map of HEF from viral particles with a matrix layer is shown with the flexibly fitted model. Carbohydrates that were modelled for five of eight potential glycosylation sites in the X-ray structure are shown at N12, N47, N130 and N381 of HEF1 and N106 of HEF2. In the map obtained by subtomogram averaging, prominent densities are observed for N12, N381, and N106. An additional density (GS1) is observed proximal to another potential site, N157. A density (GS2) near the N-terminus of HEF2 is not fully explained but may include HEF2 residues 1-3 which were not modelled in the X-ray structure. Lastly, the map shows a density bifurcation in the membrane proximal region after the C-terminus of the ectodomain model of HEF2 and is not interpreted further.

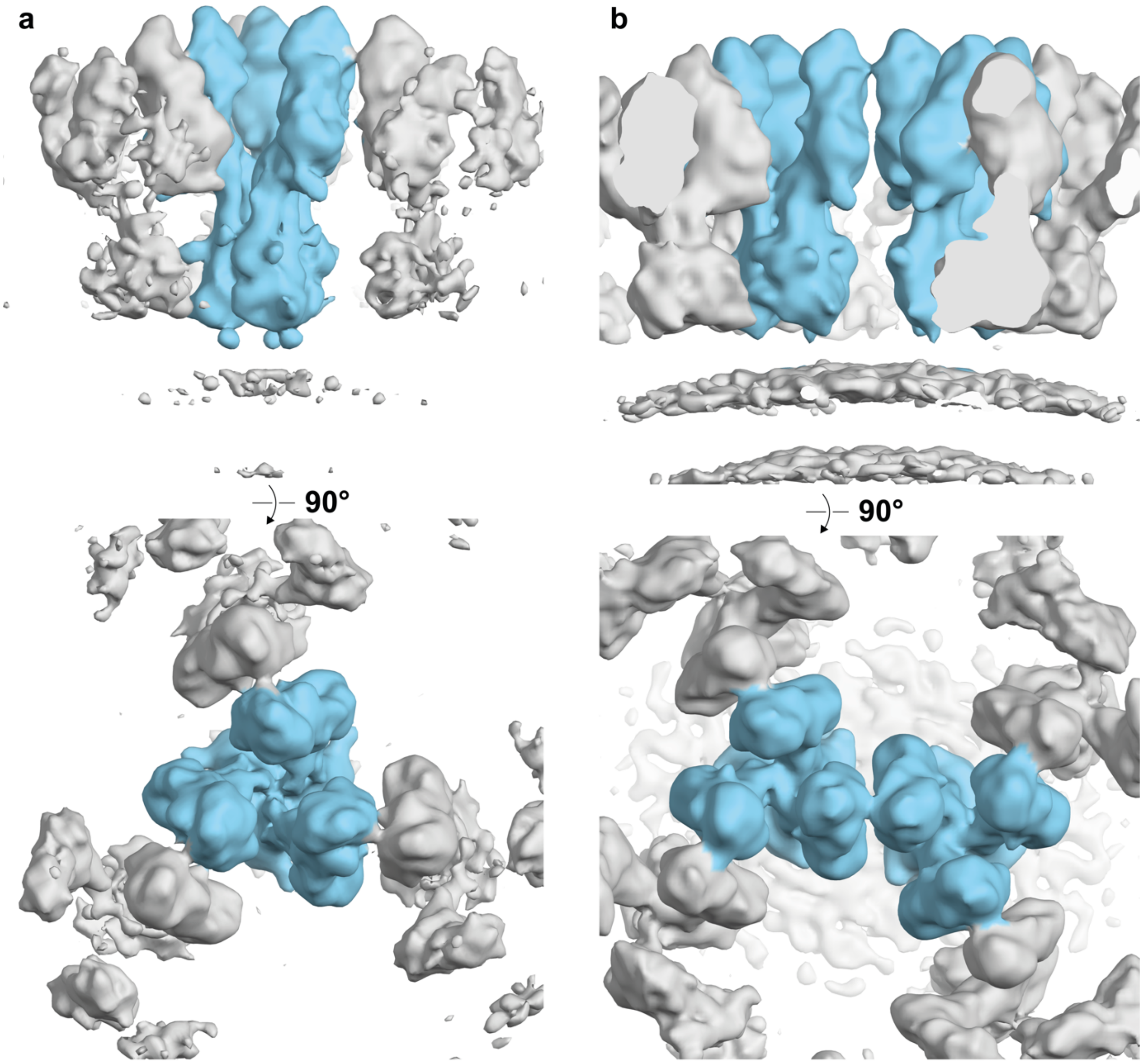

Supplementary Figure 9. *In situ* structure of HEF from particles lacking a matrix layer.

a) Side and a top view of map (10.0 Å resolution, **Supplementary Figure 4**) obtained by subtomogram averaging of HEF trimer from spherical particles that lack a matrix. Central trimer is coloured blue and neighbouring trimers and membrane are coloured grey. **b)** Side and a top view of map (11.3 Å resolution, **Supplementary Figure 4**) obtained by subtomogram averaging of two interfacing HEF trimers from spherical particles that lack a matrix.

Tomographic Data collection	Spherical virions	Filamentous virion
Microscope	Titan Krios	Titan Krios
Voltage (keV)	300	300
Nominal Magnification	64000	37000
Exposure navigation	Image shift/ Stage position	Image shift/ Stage position
Tilt range (°)	-42 to +54/-54 to +54	-54 to +54
Tilt interval	Every 3°	Every 3°
Total no. of images per tilt series	33/37	37
Electron exposure per tilt ($e^-/Å^2$)	1.57	1.36
Total electron exposure per tilt series ($e^-/Å^2$)	51.81/58.09	50.32
Exposure rate (e^-/px/s)	7.6	0.85
Detector	K2 Summit post GIF	Falcon III
Calibrated pixel size (Å)	2.2	2.23
Defocus	-2.0 to -4.5	-3.5
Automation software	Tomography (FEI)	Tomography (FEI)

Reconstruction	Trimer with matrix	Trimer without matrix	Dimer of trimers with matrix	Dimer of trimers without matrix	Filament STA*
Number of tomograms	41	36	41	36	1
Total no. extracted particles	31947	55267	31947	55267	4059
Final particle no.	14057	11081	22452	8142	375
Symmetry imposed	C3	C3	C2	C2	C2
Resolution (global)	9.2	10.0	10.7	11.3	26.2
FSC 0.5 (unmasked/masked)	14.6/11.0	17.6/14.2	15.7/12.9	21.0/20.0	29.7/29.7
FSC 0.143 (unmasked/masked)	11.0/9.2	14.2/10.0	13.8/10.7	15.2/11.3	26.2/26.2
Map to model FSC at 0.5	10.9	12.1	13.6	12.9	N/A
Applied B-factor ($Å^2$)	-500	-600	-750	-400	-500

*Subtomogram averaging

Supplementary Table 1: Tomographic data collection and subtomogram averaging reconstruction parameters
