# Supplementary material for: In situ structure and organization of the influenza C virus surface glycoprotein
Source: Nat Commun. 2021 Mar 16;12:1694. doi: 10.1038/s41467-021-21818-9 (PMC7966785; doi:10.1038/s41467-021-21818-9)
Supplement: Supplementary file 2 — Description of Additional Supplementary Files [file 41467_2021_21818_MOESM2_ESM.pdf]

Description of Additional Supplementary Files

Supplementary Movie 1: Morph of conformations (program Chimera) between pdbid 1FLC and fitted model coloured as in Figure 2.
