# Supplementary material for: In situ structure and organization of the influenza C virus surface glycoprotein
Source: Nat Commun. 2021 Mar 16;12:1694. doi: 10.1038/s41467-021-21818-9 (PMC7966785; doi:10.1038/s41467-021-21818-9)
Supplement: Supplementary file 4 — Reporting Summary [file 41467_2021_21818_MOESM4_ESM.pdf]

nature research

Corresponding author(s): Peter B. Rosenthal

Last updated by author(s): Jan 20, 2021

Reporting Summary

Nature Research wishes to improve the reproducibility of the work that we publish. This form provides structure for consistency and transparency in reporting. For further information on Nature Research policies, see our Editorial Policies and the Editorial Policy Checklist.

Statistics

For all statistical analyses, confirm that the following items are present in the figure legend, table legend, main text, or Methods section.

n/a	Confirmed	
☐	☒	The exact sample size (n) for each experimental group/condition, given as a discrete number and unit of measurement
☐	☒	A statement on whether measurements were taken from distinct samples or whether the same sample was measured repeatedly
☐	☒	The statistical test(s) used AND whether they are one- or two-sided<br>*Only common tests should be described solely by name; describe more complex techniques in the Methods section.*
☒	☐	A description of all covariates tested
☒	☐	A description of any assumptions or corrections, such as tests of normality and adjustment for multiple comparisons
☐	☒	A full description of the statistical parameters including central tendency (e.g. means) or other basic estimates (e.g. regression coefficient) AND variation (e.g. standard deviation) or associated estimates of uncertainty (e.g. confidence intervals)
☐	☒	For null hypothesis testing, the test statistic (e.g. F, t, r) with confidence intervals, effect sizes, degrees of freedom and P value noted<br>*Give P values as exact values whenever suitable.*
☒	☐	For Bayesian analysis, information on the choice of priors and Markov chain Monte Carlo settings
☒	☐	For hierarchical and complex designs, identification of the appropriate level for tests and full reporting of outcomes
☒	☐	Estimates of effect sizes (e.g. Cohen's d, Pearson's r), indicating how they were calculated

Our web collection on statistics for biologists contains articles on many of the points above.

Software and code

Policy information about availability of computer code

Data collection	CryoEM data collected using Thermo Scientific Tomography v 4
Data analysis	CryoEM data processed using following packages: RELION-3.0, CTFfind4 v.4.1.5 IMOD v.4.10.14, novaCTF (no version), SPIDER v 13.08, SciPy (no version), Dynamo v.1.1, EMAN2 v.2.31, Coot v.0.9-pre, VMD v.1.9.3, NAMD v.2.13, PHENIX v.1.17, UCSF Chimera v.1.12, DynDom v.1.0, cryoSPARC v.2.1

For manuscripts utilizing custom algorithms or software that are central to the research but not yet described in published literature, software must be made available to editors and reviewers. We strongly encourage code deposition in a community repository (e.g. GitHub). See the Nature Research guidelines for submitting code & software for further information.

Data

Policy information about availability of data

All manuscripts must include a data availability statement. This statement should provide the following information, where applicable:

- Accession codes, unique identifiers, or web links for publicly available datasets
- A list of figures that have associated raw data
- A description of any restrictions on data availability

Maps and models have been deposited in the Electron Microscopy Data Bank, http://www.ebi.ac.uk/pdbe/emdb/ (Accession Nos. EMD-10810, EMD-10811, EMD-10812, EMD-10813, EMD-10814). A model has been deposited in the Protein Data Bank, https://www.ebi.ac.uk/pdbe/ (PDB ID code: 6YI5).

Field-specific reporting

Please select the one below that is the best fit for your research. If you are not sure, read the appropriate sections before making your selection.

[x] Life sciences [ ] Behavioural & social sciences [ ] Ecological, evolutionary & environmental sciences

For a reference copy of the document with all sections, see nature.com/documents/nr-reporting-summary-flat.pdf

Life sciences study design

All studies must disclose on these points even when the disclosure is negative.

Sample size	All datasets consist of several thousands of independent images, as described in Methods. The number of images were sufficient to achieve the reported resolution, according to the most commonly reported resolution measures in cryoEM described in Rosenthal and Henderson Journal of Molecular Biology, 2003, 333, 271-745.
Data exclusions	cryoEM single particles were included and excluded using standard image processing classification techniques as described in Relion-3 (reference 34). Details of numbers of selected images are shown in Supplementary Table 1.
Replication	Structures were determined using half datasets, according to standard procedures in cryoEM. Preliminary images/tomograms of at least 3 virus preparations were consistent in low resolution visual assessment. A single grid was selected for high-resolution data collection and analysis.
Randomization	Not applicable to this study, as samples were not assigned to experimental groups and data were collected and processed according to standard techniques for cryoEM.
Blinding	Not applicable to this study, as there was no experimental group allocation in data collection and analysis.

Reporting for specific materials, systems and methods

We require information from authors about some types of materials, experimental systems and methods used in many studies. Here, indicate whether each material, system or method listed is relevant to your study. If you are not sure if a list item applies to your research, read the appropriate section before selecting a response.

Materials & experimental systems

n/a	Involved in the study	
[x]	[ ]	Antibodies
[ ]	[x]	Eukaryotic cell lines
[x]	[ ]	Palaeontology and archaeology
[x]	[ ]	Animals and other organisms
[x]	[ ]	Human research participants
[x]	[ ]	Clinical data
[x]	[ ]	Dual use research of concern

Methods

n/a	Involved in the study	
[x]	[ ]	ChIP-seq
[x]	[ ]	Flow cytometry
[x]	[ ]	MRI-based neuroimaging

Eukaryotic cell lines

Policy information about cell lines

Cell line source(s)	MDCK
Authentication	Cells were authenticated by the World Influenza Collaborating Centre at MRC NIMR (now Francis Crick Institute)
Mycoplasma contamination	Not tested for mycoplasma contamination
Commonly misidentified lines (See ICLAC register)	Commonly misidentified cell lines were not used in this study.
